# Vitamin D supplementation differentially affects seasonal multiple sclerosis disease activity

**DOI:** 10.1002/brb3.761

**Published:** 2017-07-11

**Authors:** Andrei Miclea, Marius Miclea, Maximilian Pistor, Andreas Hoepner, Andrew Chan, Robert Hoepner

**Affiliations:** ^1^ Neurological outpatient department Neurocenter Peine Peine Germany; ^2^ Medical Faculty Ruhr‐University Bochum Bochum Germany; ^3^ Banking & Finance group Michael Smurfit Graduate Business School & UCD Lochlann Quinn School of Business University College Dublin Belfield Dublin Ireland; ^4^ Department of Neurology University Hospital Bern and University of Bern Bern Switzerland

**Keywords:** 25(OH)D3, Calcitriol, relapse, seasonality, ultraviolet‐B, winter

## Abstract

**Objectives:**

Low ultraviolet‐B (UVB) radiation causes hypovitaminosis D, which is a known risk factor for multiple sclerosis (MS) and associated with MS disease activity. Our objective is to test whether vitamin D supplementation is most effective in lowering disease activity during the period of the year with low UVB radiation and consequently low serum 25‐hydroxyvitamin D_3_ (25(OH)D_3_) concentration.

**Methods:**

Retrospective analysis of medical records from our outpatient department identified 40 MS patients with available data of at least 6 months before and during oral vitamin D supplementation. Serum 25(OH)D_3_ concentration was analyzed using immunoassay. UVB radiation data were provided by the local government. Annualized and quarterly relapse rates before and during vitamin D supplementation served as outcome parameters.

**Results:**

During vitamin D supplementation (18,950 international units/week (mean, *SD* 3,397)), serum 25(OH)D_3_ concentration increased by 51 nmol/L and the UVB‐related seasonal variability in 25(OH)D_3_ levels ceased (rho = −0.13, *p* > .05). Furthermore, the annualized relapse rate decreased by approximately 50%. This was almost solely driven by the prominent reduction in the quarterly relapse rate in late winter/early spring, when 25(OH)D_3_ levels of nonsupplemented patients were the lowest.

**Conclusions:**

Our study demonstrated the modulation of seasonal MS disease activity through vitamin D supplementation. Given the prominent reduction in the quarterly relapse rate in late winter/early spring, our data indicate a beneficial effect of supplementing MS patients with vitamin D, especially during this period of the year.

## 1 INTRODUCTION

Multiple sclerosis (MS) is a chronic autoimmune disease of the central nervous system and one of the most frequent neurological disorders in young adults (Noseworthy, Lucchinetti, Rodriguez, & Weinshenker, 2000). The generally recognized MS risk factor hypovitaminosis D (Ascherio & Munger, 2007; Hanwell & Banwell, 2011) is defined as a 25‐hydroxyvitamin D (25(OH)D) concentration below 50 nmol/L and is prevalent in approximately 57% of German adults (Hintzpeter, Mensink, Thierfelder, Müller, & Scheidt‐Nave, 2008). Experimental data show that vitamin D (VD) possesses anti‐inflammatory and immunomodulatory properties through inhibition of dendritic cell differentiation, induction of T regulatory cells, blockage of Th17 differentiation, and down‐regulation of Th1 immune responses (Farias et al., 2013; Hamzaoui et al., 2014; Lemire, Archer, Beck, & Spiegelberg, 1995). Concordantly, several studies have found an inverse correlation between serum 25(OH)D concentration and disease activity in MS patients (Brola et al., 2016; Pierrot‐Deseilligny, Rivaud‐Péchoux, Clerson, de Paz, & Souberbielle, 2012; Rotstein et al., 2015; Simpson et al., 2010). These findings are supported by genetic data showing that the MS susceptibility gene HLA‐DRB1*1501 is regulated by a VD‐dependent promotor (Ramagopalan et al., 2009).

Ultraviolet‐B (UVB) radiation (290–315 nm), which decreases with greater distance from the equator, is mandatory for the conversion of 7‐dehydrocholesterol into previtamin D_3_ (Webb, Kline, & Holick, 1988). Therefore, populations of mid‐ and high‐latitude countries often lack a sufficient supply with VD and have a higher risk of developing MS (Kimlin, 2008). A retrospective data analysis conducted in France showed an association between regional variation in MS prevalence and UVB radiation (Orton et al., 2011). While UVB radiation is also thought to have a VD independent immunomodulatory effect (Breuer et al., 2014), it contributes to approximately 90% of the body's 25(OH)D supply (Federal Office for Radiation Protection (Germany), 2012). Ascherio et al. reported a 57% lower relapse rate in patients with a higher 25(OH)D concentration (Ascherio et al., 2014). Furthermore, an analysis of the international MSBase registry data demonstrated that increasing latitudes away from the equator are associated with a shorter lag between seasonal UV radiation trough and relapse peak (Spelman et al., 2014). The relapse peak was recorded in early spring (March) and the relapse trough in autumn (October), which indicate an annual cyclical pattern of MS disease activity (Spelman et al., 2014). As UVB radiation is lowest during the winter months (Webb et al., 1988) and 25(OH)D_3_ has a half‐life of approximately 15 days (Jones, 2008), VD deficiency increases during winter. Consequently, hypovitaminosis D has the most substantial impact on the immune system in late winter/early spring.

To our knowledge no other study has yet examined the interactions among seasonal variations in UVB radiation, serum 25(OH)D_3_ concentration, and MS relapse rates and the effect of VD supplementation on these associations. We aim to investigate whether MS relapse rates are higher, when UVB radiation and consequently 25(OH)D_3_ levels are low. If this increase in MS disease activity is genuinely induced by hypovitaminosis D, then VD supplementation should be most effective during this period of the year.

## 2 METHODS

### 2.1 Patient group studied

Our retrospective medical chart analysis identified 40 MS patients with an observation period of at least 6 months before and after the initiation of VD supplementation. All patients were treated between April 2010 and June 2016 at the neurological outpatient department Neurocenter Peine, in Peine (latitude 52.3, longitude 10.2), Lower Saxony, Germany. Unless contraindicated, generally all MS patients from our outpatient department are recommended to take orally 20,000 international units (IU) of VD per week. This is well below the Food and Nutrition Board's upper tolerable intake limit recommendation of 4,000 IU/day (28,000 IU/week) (Institute of Medicine, Food and Nutrition Board, 2010). Patient consultation and documentation was conducted by MM. Data were retrospectively assessed by MM and AM. VD status was determined by measuring the serum 25(OH)D_3_ concentration in a local laboratory using chemiluminescent immunoassay. If different immunotherapies were used in the predefined intervals (before and during VD supplementation), the MS therapy with the longest duration in each interval was defined as concomitant immunotherapy. Following the German MS guidelines, a relapse was defined as a worsening of clinical symptoms, which was not related to any other infectious disease or condition and lasted more than 24 hr (Gold, 2012).

### 2.2 UVB radiation

We received the local (latitude 52.2, longitude 9.1) erythemal UVB radiation (mW/m²) data from the ministry of environment, energy, and climate protection of Lower Saxony. UVB radiation was recorded from April 2010 to June 2016. Mean UVB values were calculated for each month and each quarter of the year (January–March, April–June, July–September, and October–December).

### 2.3 Endpoints

Efficacy endpoints were the individually analyzed quarterly (QRR) and annualized relapse rate (ARR). The ARR was calculated by dividing the total number of relapses by the observed time in years. Calculation of the QRR was based on the monthly relapse rates, which were calculated by dividing the cumulative number of relapses by the total number of the respective month during the entire observation period. Afterward, the monthly relapse rates of each quarter were summed.

### 2.4 Statistical analysis

We performed a descriptive analysis, which included all evaluable patients. Missing values were not imputed. The Kruskal–Wallis test was used to assess the different quarterly distributions of the year's UVB radiation. Regarding the comparison of serum 25(OH)D_3_ levels before and during VD supplementation, we followed Hintzpeter et al.'s proposal and ran a seasonal deconvolution to control for the month of sampling (Hintzpeter et al., 2008). Using Spearman's correlation coefficient, we separately investigated the patients’ data before and during VD supplementation to identify the effect of VD supplementation on the association between UVB radiation and serum 25(OH)D_3_ concentration. The Wilcoxon signed‐rank test was used to investigate the ARR and QRR before and during VD supplementation. Statistical significance was declared for *p* < .05 and illustrated in the figures using “*”. A *p*‐value < .01 was depicted as “**”. All analyses were performed using SPSS Statistics 20^®^ (IBM Corporation: Armonk, New York, USA).

### 2.5 Ethical approval

The present retrospective data analysis was in accordance with the ethic committee of the medical association of Lower Saxony, Germany.

## 3 RESULTS

**Figure 1:**
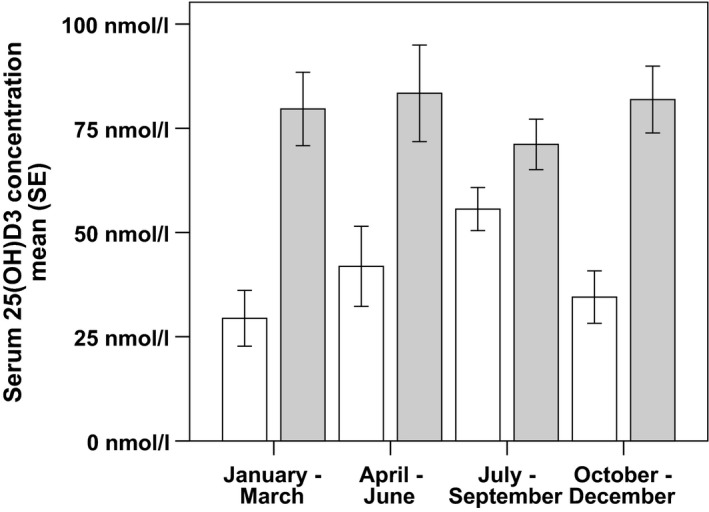
Serum 25(OH)D_3_ concentration before and during vitamin D supplementation. White bars: Before VD supplementation, grey bars: During VD supplementation. N: January–March: Before VD 12, during VD 11; April–June: Before VD 8, during VD 10; July–September: Before VD 11, during VD 18; October–December: Before VD 12, during VD 12. Abbreviations: SE, Standard error; VD, Vitamin D; 25(OH)D_3_, 25‐hydroxyvitamin D_3_

The baseline characteristics of the study population are presented in Table 1. The serum samples of 25(OH)D_3_ measurements were obtained 2 years (mean, *SD* 1) after the initiation of VD supplementation. Patients received a mean VD dose of 18,950 IU per week (*SD* 3,397), which increased the mean serum 25(OH)D_3_ concentration by 51.4 nmol/L (Table 1). The year's UVB radiation differed significantly as higher values were recorded from April to September and lower values were recorded from October to March (Figure S1). Before VD supplementation, we found a strong correlation between the monthly UVB radiation at sampling and serum 25(OH)D_3_ concentration (rho = 0.41, *p* < .01, number of 25(OH)D_3_ measurements: 47). Consequently, serum 25(OH)D_3_ levels of nonsupplemented MS patients showed seasonal variations exhibiting a peak from July to September (Figure S1), which labels the end of the period of high UVB exposure (Figure S1). This was the only period of the year during which serum 25(OH)D_3_ levels of non‐supplemented patients were above the lower limit of normal (50 nmol/L (Hintzpeter et al., 2008)). In contrast, during VD supplementation, serum 25(OH)D_3_ levels remained steadily above 70 nmol/L and the seasonal fluctuations ceased (Figure 1). The correlation between UVB radiation and serum 25(OH)D_3_ concentration was no longer significant (rho = −0.13, *p* = .33, number of 25(OH)D_3_ measurements: 62).

**Table 1:** Baseline characteristics

Baseline characteristics	Mean	*SD*	***N***
Age at MS diagnosis (in years)	33.9	11.8	40
Age at first consultation (in years)	42.1	11.0	40
Age at start of VD supplementation (in years)	44.1	11.0	40
EDSS score at start of VD supplementation	3.1	1.5	39
VD dose (in international units/week)	18,950	3,397	40
Gender	%		***N***
Female	60	—	24
MS phenotype	%		***N***
RRMS	75	—	30
PRMS	25	—	10

	Before VD	During VD	*p*‐value
Additional information			
Serum 25(OH)D_3_ levels (mean (*SD*) (in nmol/L)), *n* = 26^a^	38.7 (24.1)	90.1 (27.1)	<.001^b^
Time of follow up (mean (*SD*) (in years)), *n* = 40	2.0 (1.4)	2.6 (1.2)	n.s.^b^
Immunotherapy			
Interferons	35%	30%	n.s.^c^
No therapy	30%	22.5%	n.s.^c^
Glatiramer acetate	17.5%	20%	n.s.^c^
Natalizumab	10%	17.5%	n.s.^c^
Immunosuppressants	5%	2.5%	n.s.^c^
Glucocorticoids	2.5%	2.5%	n.s.^c^
Dimethyl fumarate	0%	5%	n.s.^c^

EDSS, Expanded disability status scale; MS, Multiple sclerosis; n.s., Nonsignificant; PR, Progressive relapsing; RR, Relapsing remitting; VD, Vitamin D; 25(OH)D_3_, 25‐hydroxyvitamin D_3_.

a Included were only patients with available 25(OH)D_3_ measurements before and during VD supplementation.

b Wilcoxon signed‐rank test.

c Chi‐square.

**Figure 2:**
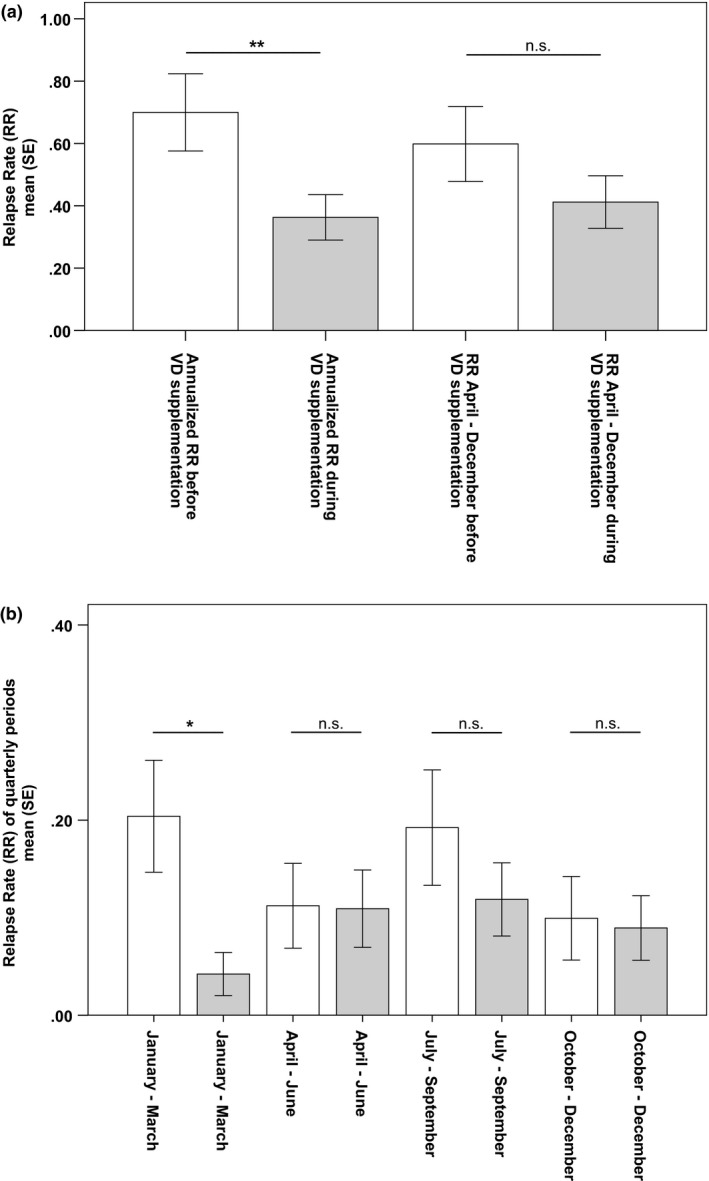
Relapse rates before and during vitamin D supplementation. (a) relapse rates with and without the first quarter. Included patients per year, before//during VD supplementation: 40//40. Statistics: Wilcoxon signed‐rank test. (b) Relapse rates of quarterly periods. White bars: Before VD supplementation. Grey bars: During VD supplementation. Included patients per quarter, before//during VD supplementation: quarter 1: 40//40, quarter 2: 40//39, quarter 3: 39//39, quarter 4: 36//39. Statistics: Wilcoxon signed‐rank test. Abbreviations: n.s., Nonsignificant; SE, Standard error; VD, Vitamin D

In comparison to before supplementation, the ARR was approximately 50% lower during VD supplementation (Figure 2a), which was driven by the significantly lower QRR in the first quarter of the year (January–March) (Figure 2b). Before VD supplementation, also the lowest serum 25(OH)D_3_ levels were measured in the period from January to March (Figure 1). By excluding this period from the relapse rate calculation, the difference in relapse rates was no longer significant (Figure 2a). A multivariate cross‐sectional linear regression analysis, which included the ARR as outcome variable and VD supplementation, immunotherapies, MS phenotype, gender, age, and disease duration as independent variables, proved that VD supplementation has a significant effect on MS disease activity (regression coefficient for VD supplementation, −0.24; 95% confidence interval, −0.61 to −0.02; *p* = .04).

## 4 DISCUSSION

Investigating the effect of VD supplementation on the interactions between seasonal variations in UVB radiation, serum 25(OH)D_3_ concentration, and MS disease activity, we demonstrated that relapse rates were higher and more subject to seasonal fluctuations before the supplementation with VD. VD supplementation had the greatest effect on relapse rates in the first quarter of the year, when UVB radiation and consequently 25(OH)D_3_ levels of nonsupplemented patients were low.

Through oral supplementation with VD (18,950 IU per week [mean, *SD* 3,397]), serum 25(OH)D_3_ levels increased significantly by approximately 51 nmol/L (Table 1). Similarly, a prospective cohort study from Finland reported an increase of 56 nmol/L after supplementing patients with 20,000 IU of VD per week for 12 months (Soilu‐Hänninen et al., 2012).

In our study cohort, the ARR decreased by approximately 50% after the initiation of VD supplementation. In comparison, Burton et al. reported a slightly lower reduction in the ARR (41%) (Burton et al., 2010). The patients included in our study were supplemented with VD regardless of their baseline serum 25(OH)D_3_ concentration. Therefore, we cannot rule out that also patients with sufficient 25(OH)D_3_ levels were enrolled. It is more likely, however, that this would have led to a reduced rather than an increased effect of VD supplementation.

The effect of VD supplementation on MS disease activity was most prominent in late winter/early spring (January–March), which is the period of the year with low UVB radiation and the lowest serum 25(OH)D_3_ concentration. Interestingly, we did not observe an increase in disease activity from October to December, when UVB radiation is low as well and 25(OH)D_3_ levels start to decline. An epidemiological study supports this finding demonstrating that the increase in relapse rate lagged 1.5 months behind the local UVB radiation (Tremlett et al., 2008). Therefore, UVB radiation of late summer might also influence relapses in the fourth quarter of the year. The approximately 15‐day half‐life of 25(OH)D_3_ might play a key role in this lag (Jones, 2008). As our work was a purely retrospective study of medical records, we cannot provide seasonal immunological data to support our explanation for the lag in relapse rates. Immunological alterations induced by hypovitaminosis D such as suppression of inflammatory cytokines, stimulation of T‐regulatory and Th2‐cell differentiation, inhibition of B‐cell development, and modulation of the Nf‐kappa‐B pathway (Szymczak & Pawliczak, 2016) might appear earlier than the clinical effects—in our case the increase in ARR. The lack of laboratory investigations is therefore the major weakness of our study and should be considered while interpreting the epidemiological data.

Before VD supplementation, patients also demonstrated a peak of relapse rates from July to September, which was not paralleled by 25(OH)D_3_ deficiency. This relapse peak points to additional triggering factors of disease activity aside from UVB radiation and serum 25(OH)D_3_ concentration. Oqawa G et al. demonstrated in a Japanese cohort of MS patients that relapse rates had two peaks over the year: one in the winter and one the in summer (Oqawa et al., 2004). The summer peak in relapse rates was explained by higher temperatures leading to immunological modulations, in particularly to a change in leukocyte nitric‐oxide production (Beenakker et al., 2001; Oqawa et al., 2004). A complementary immunological explanation from a recently conducted study are the summer troughs of melatonin production, which ultimately lead to the induction of pathogenic Th17‐cell differentiation, inhibition of protective T‐regulatory cells, and deactivation of IL‐10 promotors (Farez et al., 2015). Further studies are warranted to clarify these hypotheses as possible reasons for the increase in relapse rates during summer.

Retrospective studies are generally limited in their validity. However, we have no evidence for biases often associated with retrospective study designs, for example, selection or information bias. To check for the selection bias, we compared the baseline characteristics of included patients (≥6 months follow‐up, *n* = 40) with the baseline characteristics of all other MS patients from the outpatient department (*n* = 65). In this comparison, we did not find significant differences between both patient groups regarding age, gender, MS phenotype, ARR, and score on the expanded disability status scale. An information bias is unlikely because patient consultation and documentation was conducted by only one physician (MM). Also, a regression to the mean effect appears unlikely as the initiation of VD supplementation was not driven by disease activity, which is supported by the inclusion of 15/40 patients who were relapse free before the start of VD supplementation. Furthermore, the correlation analysis between UVB radiation and serum 25(OH)D_3_ concentration needs clarification. As indicated by the included data points, some patients had more than one observation in this correlation analysis. Consequently, some individuals possibly contributed more than others to the observed effect. However, as UVB radiation contributes to approximately 90% of the body's 25(OH)D_3_ supply (Federal Office for Radiation Protection (Germany), 2012), we conclude that seasonal UVB radiation has a stronger impact on serum 25(OH)D_3_ levels than the effects caused by each individual patient. Our conclusion is supported by a correlation analysis between the mean values of serum 25(OH)D_3_ levels and the mean concurrent UVB radiation, which precludes the inclusion of more than one observation per patient (prior VD supplementation: rho = 0.42, *p* = .01, *n* = 35; during VD supplementation: rho = −0.24, *p* = .19, *n* = 32). Lastly, immunotherapies differed between observation periods, which might have a major effect on the ARR as our outcome parameter. However, our multivariate cross‐sectional linear regression analysis proved that VD supplementation has a significant effect on MS disease activity.

Altogether, our data indicate that VD supplementation has beneficial effects on MS disease activity and that these effects are higher during seasons with low UVB radiation and consequently insufficient 25(OH)D_3_ supply. Therefore, our data argue for an intensified VD supplementation in MS patients, especially during winter and early spring. Regarding the seasonality of VD efficacy, upcoming VD supplementation studies should adjust their efficacy analysis for the different seasons of the year. Furthermore, future research should focus on the identification of additional triggering factors of MS disease activity. Ideally—as is the case with VD—these factors could be modulated therapeutically. In addition to standard medication, this would allow supportive therapies adjusted for the specific immunological demands during different seasons of the year.

## CONFLICT OF INTEREST AND SOURCES OF FUNDING STATEMENT

On behalf of all authors, the corresponding author states that there is no conflict of interest. This research received no specific grant from any funding agency in the public, commercial, or not‐for‐profit sectors.

## DISCLOSURES

A Miclea reports no disclosures. M Miclea received travel grants from Novartis, Biogen Idec, Bayer, Teva, and Merck Serono. M Pistor reports no disclosures. A Hoepner reports no disclosures. A Chan received personal compensation as a speaker or consultant for Bayer, Biogen, Genzyme, Merck, Sanofi, Roche, and Teva Neuroscience. He also received research support from Biogen, Genzyme, and Novartis. R Hoepner received research and travel grants from Novartis and Biogen Idec.

## AUTHORS’ CONTRIBUTIONS

A. Miclea: Collected the data, contributed to the design of the study, the analysis and interpretation of the data, and the writing and revision of the manuscript; M. Miclea: Collected the data, contributed to the design of the study, the interpretation of the data, and the revision of the manuscript; M. Pistor: Contributed to the interpretation of the data and the revision of the manuscript; A. Hoepner: Contributed to the interpretation of the data and the revision of the manuscript; A. Chan: Contributed to the interpretation of the data and the revision of the manuscript; R. Hoepner: Contributed to the design of the study, the analysis and interpretation of the data, and the writing and revision of the manuscript.
