# Supplementary figures and images for: Vitamin D supplementation differentially affects seasonal multiple sclerosis disease activity
Source: Brain Behav. 2017 Jul 11;7(8):e00761. doi: 10.1002/brb3.761 (PMC5561321; doi:10.1002/brb3.761)

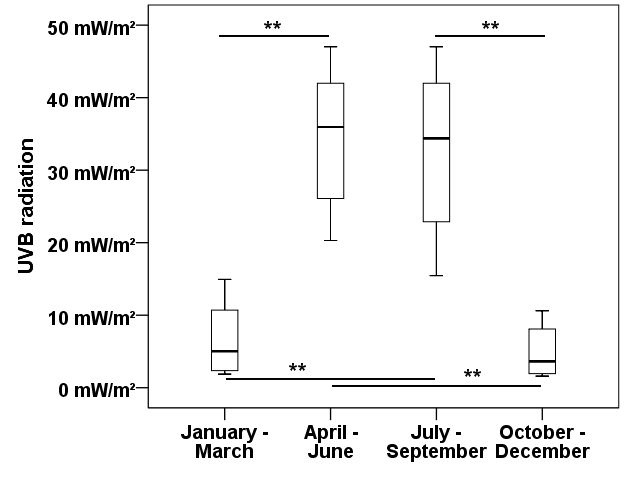

Supplement: Supplementary file 1 [file BRB3-7-e00761-s001.tif]
